# Broken Bar Fault Detection Using Taylor–Fourier Filters and Statistical Analysis

**DOI:** 10.3390/e25010044

**Published:** 2022-12-27

**Authors:** Sarahi Aguayo-Tapia, Gerardo Avalos-Almazan, Jose de Jesus Rangel-Magdaleno, Mario R. A. Paternina

**Affiliations:** 1 Digital Systems Group, National Institute for Astrophysics, Optics and Electronics, Puebla 72840, Mexico; 2 Department of Electrical Engineering, National Autonomous University of Mexico, Mexico City 04510, Mexico

**Keywords:** induction motor, broken bar, stator current, fault detection, statistical analysis, digital Taylor–Fourier transform

## Abstract

Broken rotor bars in induction motors make up one of the typical fault types that are challenging to detect. This type of damage can provoke adverse effects on the motors, such as mechanical and electrical stresses, together with an increase in electricity consumption, causing higher operative costs and losses related to the maintenance times or even the motor replacement if the damage has led to a complete failure. To prevent such situations, diverse signal processing algorithms have been applied to incipient fault detection, using different variables to analyze, such as vibrations, current, or flux. To counteract the broken rotor bar damage, this paper focuses on a motor current signal analysis for early broken bar detection and classification by using the digital Taylor–Fourier transform (DTFT), whose implementation allows fine filtering and amplitude estimation with the final purpose of achieving an incipient fault detection. The detection is based on an analysis of variance followed by a Tukey test of the estimated amplitude. The proposed methodology is implemented in Matlab using the O-splines of the DTFT to reduce the computational load compared with other methods. The analysis is focused on groups of 50-test of current signals corresponding to different damage levels for a motor operating at 50% and 75% of its full load.

## 1. Introduction

High efficiency, low cost, and low maintenance make up some of the main features of induction motors (IM) that make them the preferred machine type in the industry. For this reason, early fault detection becomes critical to schedule maintenance and prevent further damage to the motor [1].

Various factors can produce a fault in an IM, such as manufacturing defects, corrosion, overloading, and overheating [2]. The most common damages in IMs are related to bearing faults, stator faults, and broken rotor bar faults [3,4]. Among these, the broken bar damages are the most difficult to detect because this condition does not show any effect on the motor at the early stages of the fault.

Suppose the broken bar damage is not conveniently detected and treated. In that case, the unbalanced currents flowing through the rest of the rotor bars lead to thermal and mechanical stresses that may conduct the IM to complete failure. Moreover, this condition affects the operative cost of the machine, considering that this type of fault can significantly increase power consumption.

To achieve a convenient detection of broken bars, diverse techniques have been studied in recent years; some of them are based on vibration signals, some others on current analysis, and magnetic flux, among others [5,6,7,8,9,10,11]. For example, Morales-Perez et al. [5] explored the use of trained dictionaries to decompose vibration signals using the orthogonal matching pursuit algorithm. Panagiotou et al. [6] proposed the detection of broken rotor bars at steady state by analyzing stray flux signals. Garcia-Calva et al. [8] analyzed the speed signature of a faulted motor in the time-frequency domain. Overall, motor current signature analysis (MCSA) is widely used because current signals acquisition does not require additional sensors in the IM.

Previous works have studied the broken bar problem using MCSA, relying on traditional methods such as the fast Fourier transform (FFT) or the wavelet transform. For instance, da Costa et al. [12] used two diagnostic methods based on the FFT and Wavelet Transform, to compare them. García-Perez et al. [13] employed the multiple signal classification (MUSIC) method applied to current and vibration signals to identify multiple faults. Trujillo-Guardado et al. [14] proposed an algorithm for broken bar detection hinged on the use of two Taylor-Kalman filters in cascade.

For classification purposes, most of the recent works use artificial intelligence (AI) methods: Support vector machines (SVM), artificial neural networks (ANN), and convolutional neural networks, among others. For instance, Reljic et al. [15] introduced ANN and SVM to perform broken bar detection. Xiao et al. [16] utilized a recurrence quantification analysis and long short-term memory neural network to detect different faults in the IM. Valtierra-Rodriguez et al. [17] proposed using convolutional neural networks to detect broken rotor bars automatically. Kumar and Hati et al. [18] employed a dilated convolutional neural network model to detect bearing and broken rotor faults based on vibration signals. These techniques have proven high accuracy, but their main drawback is the high computational resources required for their use, which may be difficult to implement for monitoring purposes. Moreover, even with the use of AI techniques, most recent works are able to detect no less than half-broken bars.

Aiming to improve the detection rate without consuming large computational resources or requiring long computational times, the Taylor–Fourier Transform (TFT) emerges as a good option for signal processing based on its excellent filtering characteristics. In general terms, the TFT is an expansion of the Fourier subspace given by the modulation of each Fourier harmonic by a Taylor polynomial of order *K* greater than zero. The TFT has been mainly applied to power systems monitoring, and oscillatory signals analysis [19,20]. This transform delivers dynamic phasor estimation, which opens the possibility of working with transitory signals. Applying the TFT to a current signal offers the possibility to estimate mainly but not only the amplitude and phase of the signal. On the other hand, the O-splines implementation of the TFT considerably reduces the computation resources required for signal processing.

In this paper, MCSA for broken bar fault classification is implemented using Taylor–Fourier filters and taking advantage of the processing facilities given by the use of the O-splines. The feature extraction is performed based on the amplitude estimation given by the TFT. Finally, an analysis of variance is performed on each distribution to detect the damage using the Tukey procedure.

This paper is organized as follows. In Section 2, fundamentals of the TFT and its O-splines are presented, as well as the basis for MCSA and statistical analysis. Then, the proposed methodology for broken bar fault classification is given in Section 3. Results are presented in Section 4, and finally, conclusions are given in Section 5.

## 2. Fundamentals

### 2.1. Digital Taylor–Fourier Transform (DTFT)

The TFT is an expansion of the Fourier Transform. It can be seen as a Finite Impulse Response (FIR) filter bank formed by a set of maximally flat filters with less inter-harmonic interference than a Fourier Transform [21].

The signal model of the DTFT is given by

(1) $$x\left(n\right)=\sum_{h=-H}^{H}p_{h}\left(n\right)e^{j2\pi hf_{1}n},\;-C\frac{N}{2}\leq n\leq C\frac{N}{2}$$

where n=0,1,⋯,N−1 are the samples of the signal being *N* the number of samples per cycle, $p_{h}\left(n\right)=a\left(n\right)e^{j\phi (n)}$ represents the dynamic phasor, a(n) and ϕ(n) are the amplitude and phase respectively, and $f_{1}$ stands for the central frequency. *C* is the number of cycles defined as C=K+1, with *K* being the number of Taylor polynomials used to approach $p_{h}\left(n\right)$ as:

(2) $$p_{h}^{K}\left(n\right)=p_{h}\left(n_{0}\right)+\dot{p_{h}}\left(n_{0}\right)\left(n-n_{0}\right)+\cdots +p_{h}^{K}\left(n_{0}\right)\frac{{\left(n-n_{0}\right)}^{K}}{K!}$$

A least square problem appears with the inclusion of (2) into (1) and leads to a factorized form of a matrix B that includes the Taylor Y and Fourier Ω terms, respectively [19]:

(3) $$B=Y\Omega =\left(\begin{array}{cccc} I & T_{1} & \cdots & \frac{1}{K!}T{}_{1}^{K}\\ I & T_{2} & \cdots & \frac{1}{K!}T{}_{2}^{K}\\ \vdots & \vdots & \ddots & \vdots \\ I & T_{C} & \cdots & \frac{1}{K!}T{}_{C}^{K} \end{array}\right)\left(\begin{array}{cccc} W_{N} & 0 & \cdots & 0\\ 0 & W_{N} & \cdots & 0\\ \vdots & \vdots & \ddots & \vdots \\ 0 & 0 & \cdots & W_{N} \end{array}\right)$$

where $T_{C}^{K}$ are diagonal submatrices containing the sequence $t={\left[-L_{h}\;-L_{h}+1\cdots L_{h}\right]}^{T}/\left(NF_{1}\right)$ where $L_{h}=\left(L-1\right)/2$ and L=CN; $W_{N}$ is a Fourier matrix given by:

(4) $$W_{N}=\left(\begin{array}{cccccc} 1 & 1 & 1 & \cdots & 1 & \\ 1 & \omega_{N} & \omega_{N}^{2} & \cdots & \omega_{N}^{\left(N-1\right)}\\ 1 & \omega_{N}^{2} & \omega_{N}^{4} & \cdots & \omega_{N}^{2(N-1)}\\ \vdots & \vdots & \vdots & \ddots & \vdots \\ 1 & \omega_{N}^{\left(N-1\right)} & \omega_{N}^{2(N-1)} & \cdots & \omega_{N}^{{\left(N-1\right)}^{2}} \end{array}\right)$$

where $\omega_{N}=e^{j2\pi /N}$ is the harmonic phase factor for the Nth harmonic.

The solution of the least square problem leads to the expression for the analysis equation, which is the core of the filtering algorithm:

(5) $$\hat{\xi }={\tilde{B}}^{H}p,$$

where $\hat{\xi }$ encloses the Taylor–Fourier coefficients; the dual matrix, $\tilde{B}$, is defined by:

(6) $$\tilde{B}=B{\left(B^{H}B\right)}^{-1}$$

Then, considering that B=YΩ, we have the following expression [19]:

(7) $$\tilde{B}=Y{\left(Y^{H}Y\right)}^{-1}\frac{\Omega }{N}=\tilde{Y}\frac{\Omega }{N}$$

Given that Y is an invertible square matrix, its dual is the transposed inverse matrix [22], this is:

(8) $$\tilde{Y}={\left(Y^{-1}\right)}^{T}=\frac{{\left(Cof{\left(Y\right)}^{T}\right)}^{T}}{\left|Y\right|}=\frac{Cof(Y)}{\left|Y\right|}$$

where Cof(Y) and |Y| are the matrix of cofactors and the determinant of the matrix Y, respectively.

Finally, the amplitude estimation $\hat{\alpha }$ is obtained from the Taylor–Fourier coefficients $\hat{\xi }$ through:

(9) $$\hat{\alpha }=2\left|\hat{\xi }\right|$$

### 2.2. O-Splines

In this section, the *K*th O-spline and its first derivatives are calculated in closed-form based on the expression stated in (8), where the inverse matrix of Y is calculated. Its rows are concatenated in one vector ${\tilde{B}}_{0}^{K}$. For amplitude and phase estimation, only the first vector of the dual matrix ${\tilde{B}}_{0}^{\left(K\right)}$ is required.

Hence, for K=0 we need one observation cycle with $t_{1}=t_{\left[-\frac{T_{1}}{2},\frac{T_{1}}{2}\right)}$; in this case $B_{0}=1$ and consequently $\tilde{B_{0}^{0}}=1$. The O-spline is a rectangular pulse given by [23]:

(10) $${\tilde{B}}_{0}^{\left(0\right)}\left(t\right)=\left\{\begin{array}{ccc} 1 & for & -\frac{T_{1}}{2}\leq t\frac{T_{1}}{2},\\ 0 & \; & otherwise. \end{array}\right.$$

For K=1 two cycles are required with $t_{1}=t_{\left[-T_{1},0\right)}$ and $t_{2}=t_{\left[0,T_{1}\right)}=t_{1}+T_{1}$; then, we have

(11) $$B_{o}^{\left(1\right)}=\left(\begin{array}{cc} 1 & t_{1}\\ 1 & t_{2} \end{array}\right)$$

with $|B_{o}^{\left(1\right)}|=t_{2}-t_{1}=T_{1}$. Then:

(12) $${\tilde{B}}_{o}^{\left(1\right)}=\frac{\left(\begin{array}{cc} t_{2} & -1\\ -t_{1} & 1 \end{array}\right)}{T_{1}}=\left(\begin{array}{cc} u_{1}+1 & -F_{1}\\ -(u_{2}-1) & +F_{1} \end{array}\right)$$

where $u_{n}$ represents the normalized time $u=t_{n}/T_{1}$. The columns of the matrix $B_{o}^{\left(1\right)}$ describe a triangular pulse:

(13) $${\tilde{B}}_{o}^{\left(1\right)}\left(u\right)=\left\{\begin{array}{ccc} u+1 & for & -1\leq u<0,\\ 1-u & for & 0\leq u<1,\\ 0 & \; & otherwise. \end{array}\right.$$

This process is repeated with the consecutive values of *K*; however, it is noteworthy to point out that only the first two polynomials are needed to obtain the rest of the elements of the matrix ${\tilde{B}}_{0}^{\left(K\right)}$ by recursive differentiation.

### 2.3. Motor Current Signature Analysis (MCSA)

The MCSA method has been applied in several works to detect different types of faults in IMs. Morever, fault detection systems are typically based on the online monitoring of stator current signals [3]. In the particular case of broken bars ($f_{bb}$), this type of fault induces a spurious component with a frequency pattern given by

(14) $$f_{bb}=f_{1}\left(1\pm 2ks\right)$$

where $f_{1}$ is the supply frequency, *k* is the number of the harmonic, and *s* represents the slip of the motor [24]. Considering this feature, the localization of the fault component is known; however, it is important to note that this component is very small compared to the fundamental frequency. Moreover, it lies very close to the supply frequency, making its detection challenging.

### 2.4. Analysis of Variance and Tukey Procedure

The analysis of variance (ANOVA) allows testing equality among different data distributions by comparing the variance between their distributions relative and the variance within distributions. This test is used in several fields, such as agricultural experiments and medical research. Depending on the type of relationship among groups or distributions, a certain model of ANOVA is applied. For instance, to determine if any differences exist in the means of 3 or more groups, the one-way ANOVA test is the most common method [25].

The first step for applying this method consists in formulating the null hypothesis, which normally is “the population means of the groups are equal” or $H_{0}:\mu_{1}=\mu_{2}=\mu_{3}$, whether the alternative hypothesis would be “at least one of the group means is different” or $H_{1}:\mu_{1}\neq \mu_{2}$ or $\mu_{1}\neq \mu_{3}$ or $\mu_{2}\neq \mu_{3}$. If at least one group differs, the null hypothesis can be rejected [25].

The core of the ANOVA test is the *F* calculation, which is comprised of the variance ratios [26]:

(15) $$F=\frac{\mathrm{Between}\;\mathrm{groups}\;\mathrm{variance}}{\mathrm{Within}\;\mathrm{groups}\;\mathrm{variance}}=\frac{SSR_{n}\left(t\right)/\left(k-1\right)}{SSE_{n}\left(t\right)/\left(n-k\right)}$$

where $SSR_{n}\left(t\right)$ and $SSE_{n}\left(t\right)$ given by

(16) $$SSR_{n}\left(t\right)=\sum_{i=1}^{k}n_{i}{\left({\bar{X}}_{i}\left(t\right)-\bar{X}\left(t\right)\right)}^{2}$$

and

(17) $$SSE_{n}\left(t\right)=\sum_{i=1}^{k}\sum_{J=1}^{n_{i}}{\left(X_{ij}\left(t\right)-{\bar{X}}_{i}\left(t\right)\right)}^{2}$$

represents the sum of squares between groups and within groups, respectively, and $\bar{X}$ and ${\bar{X}}_{i}\left(t\right)$ represent the mean of all groups and the mean of each group, which are expressed as follows:

(18) $$\bar{X}\left(t\right)=\frac{1}{n}\sum_{i=1}^{k}\sum_{j=1}^{n_{i}}X_{ij}\left(t\right)$$

(19) $${\bar{X}}_{i}\left(t\right)=\frac{1}{n_{i}}\sum_{j=1}^{n_{i}}X_{ij}\left(t\right),$$

with i=1,⋯,k, being *k* the number of groups and *n* the number of observations of the group.

A large value of *F* provides evidence to reject $H_{0}$. Thus, a subsequent method for multiple comparisons can be applied [27]. A typical table of results for an ANOVA test includes the sum of squares, the mean sum of squares, *F*, and *P* calculated between groups and within groups.

Usually, ANOVA is followed by another technique to identify the pattern of differences in the distributions. Often, this analysis consists of pairwise comparisons between the means to determine if there is a significant difference. The Tukey test is one popular method to perform this comparison.

This test is based on the computation of *Q*, which is a criterion that evaluates the difference between the means of two groups [28].

(20) $$Q=\frac{M_{i}-M_{j}}{\sqrt{SSE_{n}\left(\frac{1}{S}\right)}}$$

where $M_{i}$ and $M_{j}$ are the mean of the groups being compared, $SSE_{n}$ is the sum of squares within groups, and *S* is the number of observations per group. This value is then compared against a $Q_{critical}$ value from a table, which depends on the degrees of freedom, the number of means being tested, and the significance level α [28].

## 3. Proposed Methodology

**Figure 1:**
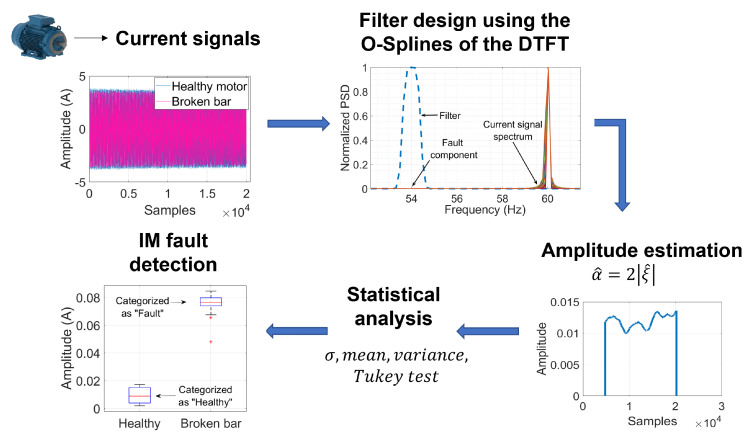
Flowchart of the proposed methodology for broken bar detection in IM.

The diagram of Figure 1 illustrates the steps that structure the proposed methodology for broken bar detection using the DTFT. First, current signals for different health conditions of the motor need to be acquired. Then, the characteristics of the Taylor–Fourier filters can be selected to perform an amplitude estimation in a frequency of interest. Afterward, a statistical analysis is performed over each group of amplitude estimations. Then the variance analysis is carried out. Hence, discrimination between healthy and damaged rotor bars can be conducted, resulting in the detection of the fault. Next, each process is explained.

### 3.1. Current Signals

These signals are associated with different damage levels in the rotor bar. Typically, the analysis is focused on one broken bar, half broken bar, and so on; the healthy motor signal also needs to be acquired. These signals do not require any pre-processing and are analyzed according to the sampling frequency at which they were acquired.

In the presence of a level of damage in the bars, spurious components typically appear around the fundamental frequency and its harmonics, as stated by the theory in Section 2.3. According to this expected location of the fault component, the central frequency $f_{c}$ of the filter is selected.

### 3.2. Filter Design

The design’s parameters need to be selected according to the characteristics of the current signal. The first important parameter is the central frequency $f_{c}$, which is selected in the estimated location of the fault to prevent any attenuation of the signal.

Another important feature is the bandwidth of the filter $f_{bw}$, which should be selected in a way that the undesirable energy peaks in the analysis are not considered. For example, a bandwidth narrow enough for analyzing a spurious component related to a broken bar fault will let out the energy of the supply frequency. In this way, the filtered signal will preserve only the features of the faulty component.

**Figure 2:**
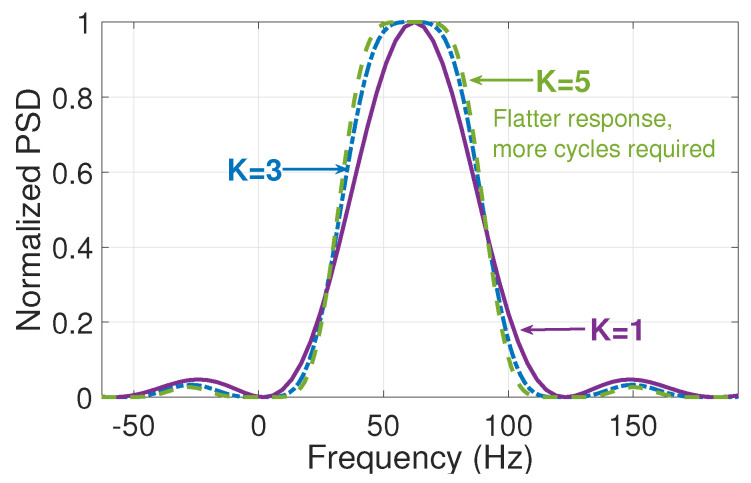
Filter response according to the value of *K* with a central frequency of 60 Hz.

Finally, the number of Taylor polynomials (*K*) is another important design parameter, considering that as this number increases, it is necessary to analyze more windows in the time domain. Still, at the same time, the filter response around the frequency of interest tends to be flatter and, therefore, more likely to be an ideal filter [29]. As can be observed from the normalized power spectral density (PSD) in Figure 2, the filter corresponding to K=5 presents a flatter response and better harmonic rejection than, for example, the one with K=1, Nonetheless, the first one requires more cycles of the signal to implement it.

Once the filter is designed, a sliding window process of size L=C×N is performed on the signal, and a single value is taken at the center of each window, forming the filtered signal. It is important to consider that the filtering results will have a delay corresponding to half of the number of cycles *C* required for the analysis; remember that this is given by selecting the value of *K*.

### 3.3. Amplitude Estimation

After the filtering process, the amplitude estimation can be obtained using (9). A single amplitude value is taken at each processed signal, forming a vector of size *M*, where *M* is the number of measurements for a certain case of damage in the bar.

### 3.4. Statistical Analysis

**Figure 3:**
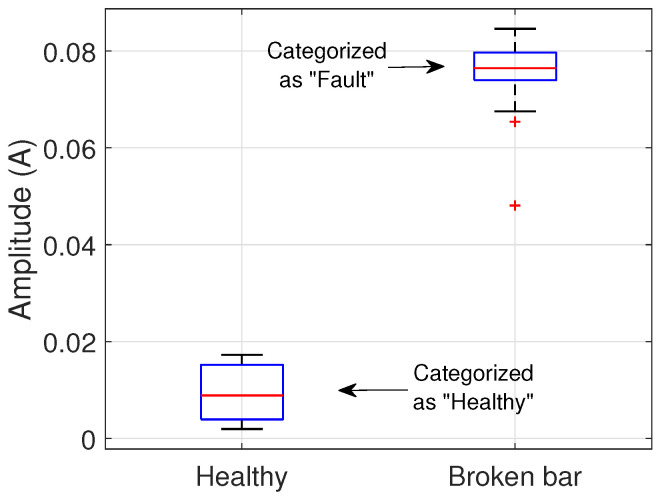
Example of classification using statistical distribution.

The variance analysis is performed, and if the null hypothesis is successfully rejected, the Tukey procedure is applied to detect the damage in the rotor’s bar. This can be achieved if the damaged groups differ significantly from the healthy group. An exaggeration of the expected behavior of the groups is depicted in Figure 3, where the broken bar induces components that grow in amplitude according to the severity of the damage; the depicted distributions fulfill the 3 standard deviations (σ) criteria of the Chebyshev theorem.

## 4. Experimental Results

**Figure 4:**
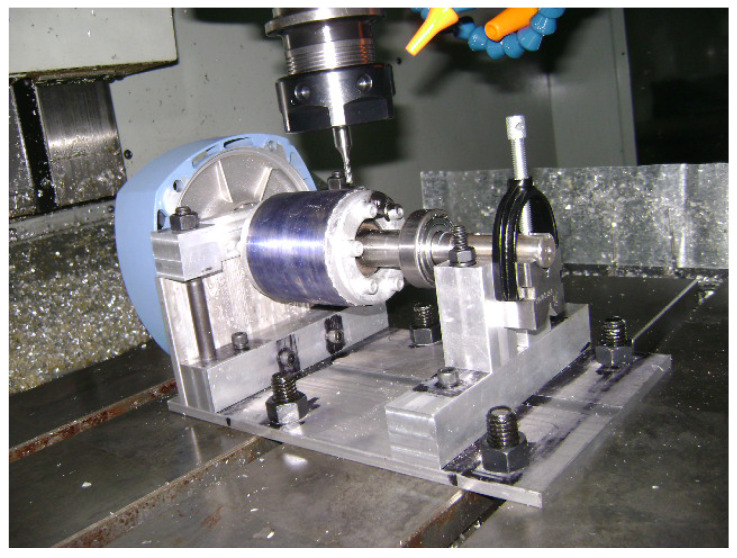
Rotor bar drilling to create the damage levels.

**Figure 5:**
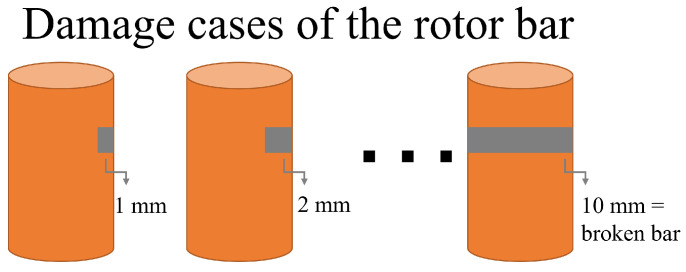
Bar damage levels under analysis.

This work analyzed 10 different damage levels for an IM operating under load conditions of 75% and 50%. Each damage level was artificially created by drilling a hole in the bar, as can be observed in Figure 4, starting from 1 mm hole perforation and increasing by 1 mm until reaching a completely broken bar, as illustrated in Figure 5. The experimental test was carried out through a three-phase IM WEG 00118ET3EM143TW, 1HP, 1800RPM, 220 VAC/60 Hz, and 2.98 A; the data was acquired at a sampling frequency of 3200 Hz. For each case of damage, 50 steady-state current measurements were analyzed in this work. For the ANOVA, a significance level of α=0.01 was selected.

**Figure 6:**
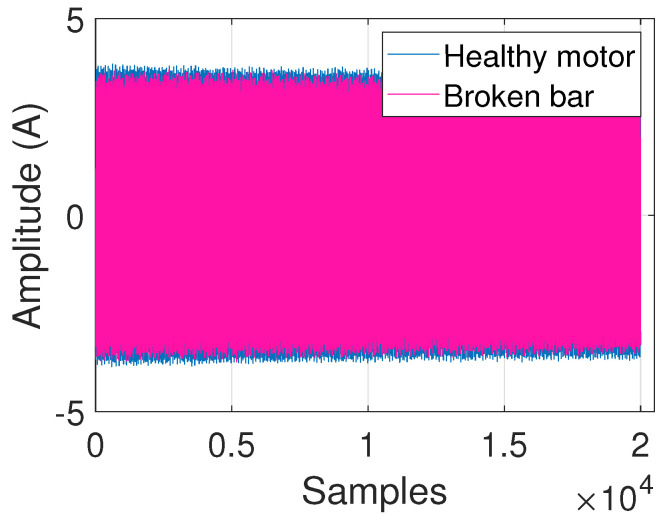
Current signals of healthy and broken bar IM.

Current signals of the healthy motor and broken bar IM illustrated in Figure 6 show that the effect of this fault is hardly noticeable in the time domain. Thus, the analysis is focused on its frequency spectrum.

**Figure 7:**
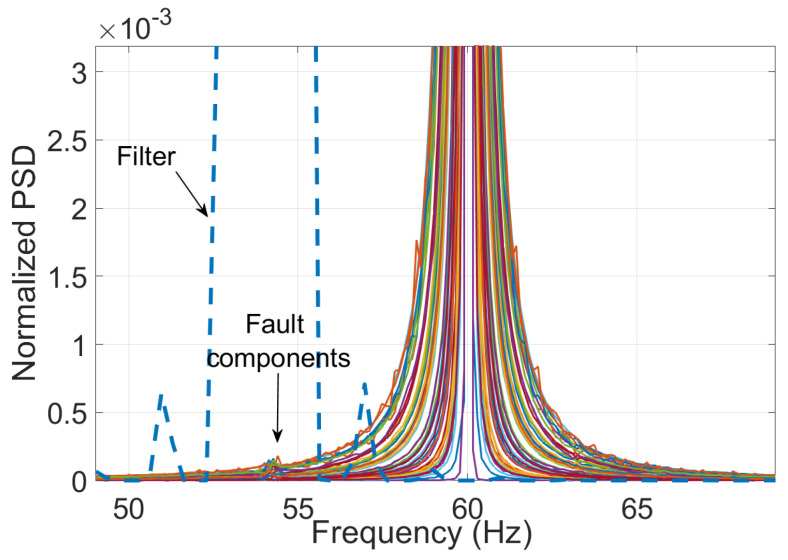
Frequency spectra of the signals for a half-broken bar IM.

**Figure 8:**
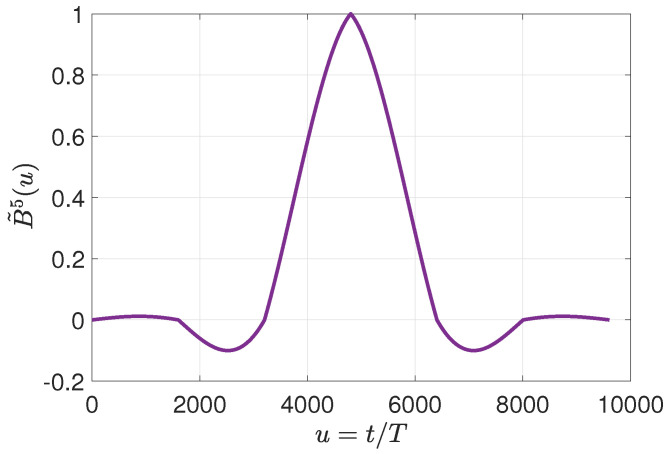
O-spline for K=5.

Figure 7 depicts the frequency spectra of 50 signals corresponding to a half-broken bar condition. Fault-related components were identified around 54 Hz. Thereby, the central frequency $f_{c}$ of the filter was selected accordingly. Continuing with the parameters for the filter design, a value of K=5 was selected for all study cases, given that the filter response is flatter enough without requiring larger segments of the signal for the analysis. This selection implies a delay in the results of 3 cycles of the signal sampled at 3200 Hz. Then, the associated O-spline is depicted in Figure 8.

**Figure 9:**
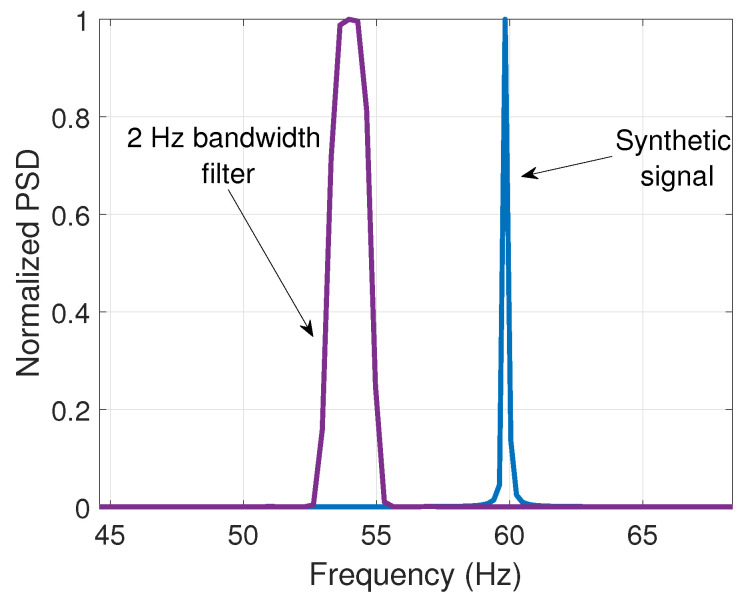
Filter with $f_{c}=$ 54 Hz and a 60 Hz synthetic signal.

It is worth noticing from Figure 7 that the spurious component represents a very small part of the signal’s energy. Besides, it lies near the center frequency of 60 Hz. For this reason, a bandwidth of 2 Hz was selected for the filters to avoid including other components that are irrelevant to the analysis. To illustrate this, Figure 9 shows the same 2 Hz bandwidth filter with a $f_{c}=$ 54 Hz and the frequency spectrum of a synthetic 60 Hz signal to remark that the reduced bandwidth excludes the energy of the fundamental component.

**Figure 10:**
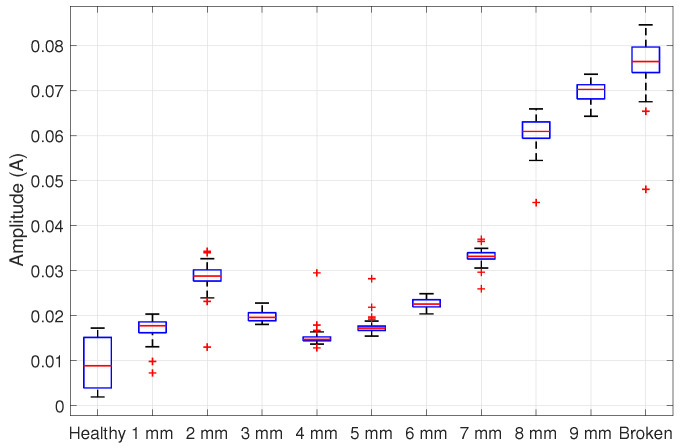
Boxplot of the fault amplitudes’ distribution data in a 75% loaded IM.

ANOVA results based on the amplitude estimations of Figure 10 are given in Table 1. The value of *p* < 0.01 rejects the null hypothesis, allowing us to continue with the Tukey procedure.

On the other hand, the Tukey test results are summarized in Table 2, suggesting that the detection is achieved when the broken bar is greater or equal to 1 mm, with a 99% of confidence level. Remember that the groups significantly differ when $Q>Q_{critical}$.

**Figure 11:**
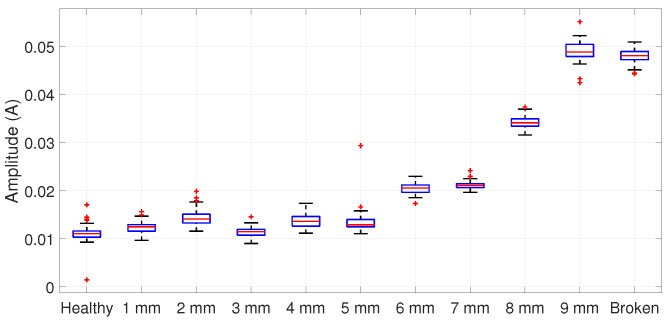
Statistical distribution for a 50% loaded motor centered at 56 Hz.

For a 50% load condition, the spurious component appears around 56 Hz; then, the filter is centered at this frequency, similar to the one presented in Figure 7. The ANOVA is applied to the amplitude distributions in Figure 11, resulting in Table 3, which allows the rejection of $H_{0}$. Hence, the Tukey test results in an incipient detection starting at 1 mm, as summarized in Table 4.

### Comparison Using Fourier filters

**Figure 12:**
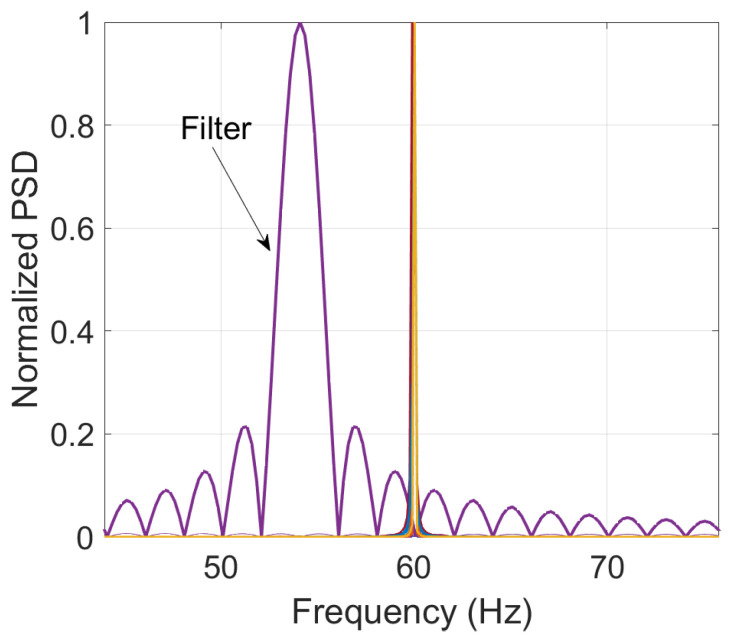
Fourier filter response.

To showcase the improvement using Taylor–Fourier filters, the proposed methodology is repeated but using a value of K=0, i.e., Fourier filters. As illustrated in Figure 12, the filter exhibits a poor harmonic rejection, giving as a result, the inclusion of undesired energy in the analysis.

**Figure 13:**
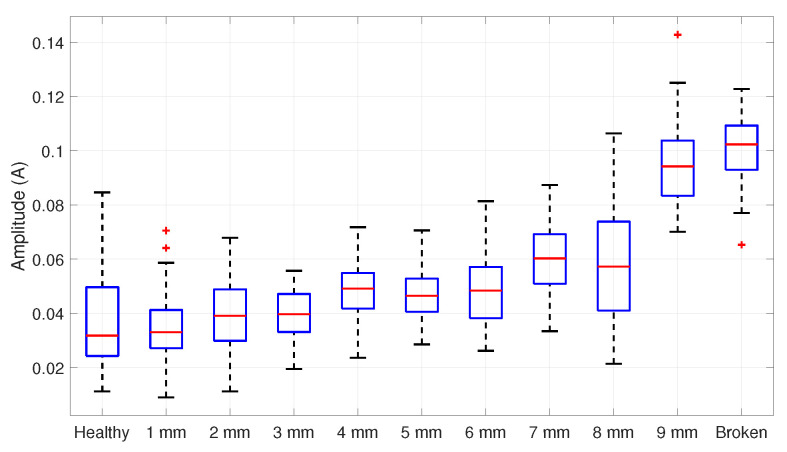
Statistical distribution for a 75% loaded motor using a Fourier filter centered at 54 Hz.

**Figure 14:**
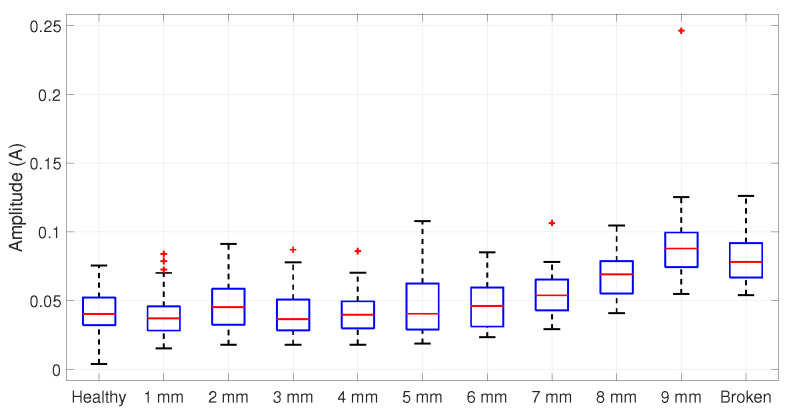
Statistical distribution for a 50% loaded motor using a Fourier filter centered at 56 Hz.

Results for the ANOVA based on the distributions of Figure 13 and Figure 14 are summarized in Table 5 and Table 6, respectively. Both cases pass the ANOVA (P <α). Nevertheless, the Tukey test results in Table 7 and Table 8 reveal that the detection is achieved at higher damage levels than Taylor–Fourier filters. In the case of 75% of load, the start of the detection goes from 1 mm to 4 mm; meanwhile, for the 50% load condition, the detection decreases from 1 mm to 8 mm.

These results demonstrate that Taylor–Fourier filters effectively detect damages in the bars of the rotor at earlier stages.

## 5. Conclusions

A methodology for early IMs broken bar detection based on the DTFT was presented and demonstrated. A total of 1100 current signals were analyzed, considering scenarios going from a healthy bar to a broken bar. For both load conditions, the Tukey test allowed an incipient detection of 1 mm damage in the bar with a confidence level of 99%; for the 75% loaded motor, only the 3 mm case could not be detected. Although the median value for the 3 mm distribution is larger than that for the healthy bar, the data dispersion tends to have smaller amplitude values. This drawback can be attributed not to the 3 mm case but to the healthy bar distribution, whose current signals presented a slight damping that tends to increase its estimated amplitude. However, the signals were purposefully not preprocessed to assess the reach of the proposed method for early fault detection. Overall, it is considered that the DTFT implementation for current signal analysis was successfully carried out to perform early fault detection.

The use of the O-splines of the DTFT enabled smooth filtering without an increase in the processing time. This is an important advantage regarding future implementation in an FPGA. In addition, the estimated fault amplitude was the only feature used for detection, which is a straightforward calculation due to the use of the O-splines.

In future works, we are interested in extracting more features with the TFT, such as the phase of the signals, in performing a classification of the damage levels in the rotor bar. Besides, this system coded in Matlab will be traduced to its equivalent in a hardware description language (VHDL) to implement it in an FPGA, aiming to assess the system’s performance for an IM operating under real circumstances.

## Figures and Tables

**Table 1:** ANOVA results for 75% loaded motor.

	Between Groups	Within Groups	Overall
**Sum of squares**	0.2828	0.0055	0.2883
**Degrees of freedom**	10	539	549
**Mean sum of squares**	0.0283	1.03 × $10^{-5}$	
**F**	2758.8433		
**P**	0		

**Table 2:** Tukey test results for 75% loaded motor, $Q_{critical}=5.227$.

Comparison	Q	Results
**Healthy vs. 1 mm **	17.2663	Significantly different
**Healthy vs. 2 mm**	42.4258	Significantly different
**Healthy vs. 3 mm**	22.8229	Significantly different
**Healthy vs. 4 mm**	12.6416	Significantly different
**Healthy vs. 5 mm**	17.7610	Significantly different
**Healthy vs. 6 mm**	29.2188	Significantly different
**Healthy vs. 7 mm**	52.2714	Significantly different
**Healthy vs. 8 mm**	113.1296	Significantly different
**Healthy vs. 9 mm**	113.2316	Significantly different
**Healthy vs. broken**	147.8344	Significantly different

**Table 3:** ANOVA results for 50% loaded motor.

	Between Groups	Within Groups	Overall
**Sum of squares**	0.1032	0.0014	0.1046
**Degrees of freedom**	10	539	549
**Mean sum of squares**	0.0103	2.60 × $10^{-6}$	
**F**	3968.8528		
**P**	0		

**Table 4:** Tukey test results for 50% loaded motor, $Q_{critical}=5.227$.

Comparison	Q	Results
**Healthy vs. 1 mm**	5.9463	Significantly different
**Healthy vs. 2 mm**	14.95354	Significantly different
**Healthy vs. 3 mm**	1.3155	Not significantly different
**Healthy vs. 4 mm**	11.6383	Significantly different
**Healthy vs. 5 mm**	10.7525	Significantly different
**Healthy vs. 6 mm**	41.5717	Significantly different
**Healthy vs. 7 mm**	44.2730	Significantly different
**Healthy vs. 8 mm**	101.2017	Significantly different
**Healthy vs. 9 mm**	166.3308	Significantly different
**Healthy vs. broken**	161.2966	Significantly different

**Table 5:** ANOVA results for 75% loaded motor using a Fourier filter.

	Between Groups	Within Groups	Overall
**Sum of squares **	0.2515	0.1038	0.3553
**Degrees of freedom**	10	539	549
**Mean sum of squares**	0.0251	0.0002	
**F**	130.5811		
**P**	5.6 × $10^{-137}$		

**Table 6:** ANOVA results for 50% loaded motor using a Fourier filter.

	Between Groups	Within Groups	Overall
**Sum of squares **	0.1482	0.1810	0.3292
**Degrees of freedom**	10	539	549
**Mean sum of squares**	0.0148	0.0003	
**F**	44.1252		
**P**	$9.48\times 10^{-64}$		

**Table 7:** Tukey test results for 75% loaded motor using a Fourier filter, $Q_{critical}=5.227$.

Comparison	Q	Results
**Healthy vs. 1 mm **	1.3594	Not significantly different
**Healthy vs. 2 mm**	1.3737	Not significantly different
**Healthy vs. 3 mm**	1.5214	Not significantly different
**Healthy vs. 4 mm**	5.6934	Significantly different
**Healthy vs. 5 mm**	4.7304	Not significantly different
**Healthy vs. 6 mm**	6.2172	Significantly different
**Healthy vs. 7 mm**	11.3767	Significantly different
**Healthy vs. 8 mm**	10.4901	Significantly different
**Healthy vs. 9 mm**	29.3997	Significantly different
**Healthy vs. broken**	32.3060	Significantly different

**Table 8:** Tukey test results for 50% loaded motor using a Fourier filter, $Q_{critical}=5.227$.

Comparison	Q	Results
**Healthy vs. 1 mm **	0.9307	Not significantly different
**Healthy vs. 2 mm**	2.2843	Not significantly different
**Healthy vs. 3 mm**	0.1505	Not significantly different
**Healthy vs. 4 mm**	0.0857	Not significantly different
**Healthy vs. 5 mm**	2.6871	Not significantly different
**Healthy vs. 6 mm**	2.0041	Not significantly different
**Healthy vs. 7 mm**	4.8410	Not significantly different
**Healthy vs. 8 mm**	10.3565	Significantly different
**Healthy vs. 9 mm**	18.6478	Significantly different
**Healthy vs. broken**	14.5947	Significantly different

## Data Availability

The data presented in this study are available on request from the corresponding author. The data are not publicly available due to privacy.

## Abbreviations

The following abbreviations are used in this manuscript:

IM	Induction motors
MCSA	Motor current signature analysis
FFT	Fast Fourier transform
AI	Artificial intelligence
SVM	Support vector machines
ANN	Artificial neural networks
TFT	Taylor–Fourier transform
DTFT	Digital Taylor–Fourier transform
FIR	Finite impulse response
MCSA	Motor current signature analysis
ANOVA	Analysis of variance
PSD	Power spectral density
